# Prenatal screening and diagnosis of genetic abnormalities: SEGO, SEQC^ML^, AEDP consensus recommendations

**DOI:** 10.1515/almed-2020-0043

**Published:** 2020-07-27

**Authors:** Belén Prieto, Begoña Adiego, Javier Suela, Inmaculada Martín, Belén Santacruz, Javier García-Planells, Mar Gil, Concepción González, Eva Barrenechea

**Affiliations:** Department of Clinical Biochemistry, Hospital Universitario Central de Asturias, Department of Biochemistry and Molecular Biochemistry, University of Oviedo, Avda Roma s/n – 33010 Oviedo, Spain; SEQC^ML^ Spanish Society of Laboratory Medicine, Barcelona, Spain; Unit of Gynecology and Obstetrics, Hospital de Móstoles, Universidad Juan Carlos I, Madrid, Spain; SESEGO Group of Ultrasound – Spanish Society of Gynecology and Obstetrics, Barcelona, Spain; Laboratory of Genomics, NIMGenetics, Madrid, Spain; AEDP Spanish Association of Prenatal Diagnosis, Barcelona, Spain; Department of Clinical Biochemistry Hospital Son Espases, Palma de Mallorca, Spain; Unit of Gynecology and Obstetrics, Hospital Universitario de Torrejón, Madrid, Spain; Instituto de Medicina Genómica, IMEGEN, Valencia, Spain; Department of Clinical Analysis Hospital Universitario Virgen de la Macarena, Seville, Spain; Department of Clinical Analysis, Hospital Universitario de Galdakao, Galdakao, Spain

**Keywords:** circulating cell-free DNA, combined screening, invasive tests, nuchal translucency

## Abstract

In this paper, the scientific societies SEGO, SEQC^ML^ and AEDP provide a series of consensus-based recommendations for prenatal screening and diagnosis of genetic abnormalities. A set of evaluation indicators are also proposed as a means to improve the quality of the biochemical, ultrasound, and genetic processes involved in prenatal screening and diagnosis of genetic anomalies. Some recommendations are also proposed in relation to invasive prenatal diagnostic procedures, more specifically regarding sample collection and genetic testing. The purpose of this proposal is to unify performance criteria and quality indicators at national level, with audits performed on a regular basis. It is strongly recommended that a national prenatal screening strategy be established and provided with the resources necessary to evaluate the performance of quality indicators and diagnostic procedures under the supervision of health authorities. Protocols should be revised on a regular basis to consider the incorporation of new cost-effective technologies.

## Part I. First-trimester screening for genetic abnormalities

### Biochemical process

#### Pre-analytical recommendations

##### Information for pregnant women and healthcare professionals

All pregnant women must be informed of the benefits and limitations of undergoing prenatal screening for fetal aneuploidy and provide prior informed consent [1].

Informed consent must be obtained (generally orally) by the professional offering the screening test, who is also responsible for informing the patient of:

– her rights
– what fetal screening involves and its voluntary nature
– the alternatives of action in case a high-risk result is obtained.

The clinical laboratory specialist will establish:

– sample collection and transport requirements
– the data required for biochemical testing and the interpretation of results
– preanalytical criteria for sample refusal
– other data of interest (turnaround time, method of delivery of test results…)

##### Pregnancy details

Along with data of the patient and the date of collection, the laboratory specialist needs to know the age and weight of the patient and the estimated gestational age to identify potential critical values in biochemical markers and evaluate recruitment adequacy.

Some adjustment factors must be introduced in the risk assessment program, such as number of fetuses and chorionicity (in twin pregnancy), ethnicity, assisted reproduction, insulin-dependent diabetes, smoking, and history of previous aneuoploidy.

##### Sample collection

The blood sample collected by venipuncture will be identified unequivocally with at least two unequivocal identifiers.

##### Stability and transport

Serum must be separated and stored at 4 °C for later testing, preferably within 72 h of collection. For longer storage periods, especially if samples are received beyond the recommended timeframe (≤24 h), serum will be frozen at −20 °C. Repeated freezing and thawing must be avoided [2].

##### Custody

In view of analyte stability, it is recommended that an aliquot of the samples be stored at −20 °C for one year, to meet claims or requests for result verification in the future.

According to UNE-EN ISO 15189:2013 standards, test protocols, sample and test records (including calibration, quality control, and results, among others), and certificates of laboratory quality and competence in the measurement of markers must be stored for a period of five years.

#### Recommendations for analysis

##### Methods and reagents

Reagents for the determination of biochemical markers in serum, pregnancy-associated plasma protein-A (PAPP-A) and the free β-human chorionic gonadotropin (free β-hCG) must bear the CE mark of approval for testing for Down’s syndrome or trisomy 21 (T21), have proven experience and have calculated median values according to gestational age. The long-term stability of reagents reduces the impact of inter-batch variations.

There are protocols for certification of analytical platforms, reagents, and computer programs for prenatal risk assessment [2], [3], [4]. At present, the *Fetal Medicine Foundation* algorithm is supported by all analytical platforms in the market, except for DPC Immulite 2000 [5].

It is recommended to use the platforms with the lowest analytical imprecision to ensure that imprecision does not exceed 10% at clinical decision thresholds (1/250 or 1/270 risk) [6].

##### Calibration standards

It is recommended to use reagents produced in accordance with European directive IVD 98/79/EC and ISO17511:2003 that are standardized against international reference materials, with traceability to the WHO IRP 75/551 and WHO RR 99/650 standards for free βhCG and WHO IRP 78/610 for PAPP-A. Test results should be expressed as UI/L or mUI/mL.

##### Analytical quality assurance

It is essential to use validated internal quality control materials from suppliers other than the manufacturer of the test used in the laboratory. These control materials must have a long expiration date and long-term stability to be able to evaluate and minimize inter-batch variability.

The analytical quality control protocol must include:

– type and frequency of control measurements
– limit of tolerance
– calibration protocol and corrective measures.

In each analytical series, it is recommended to run control samples at three concentrations per analyte according to the expected concentrations for the corresponding gestational stage. Optimal analytical imprecision is attained with between-day coefficients of variation below 3.5% [5].

In Europe, the most popular cross-comparison prenatal screening program, UK-NEQAS, analyzes the data obtained on a monthly basis and provides an annual report containing not only bias values and imprecision in the measurement of biochemical markers, but also an evaluation of the estimated risk for each sample [4], [7].

##### Conversion of results to multiples of the median (MoM)

Results must be expressed as MoM for the gestational age and adjusted for the correction factors detailed in section Pregnancy details, as they have a significant impact on the MoM of biochemical markers [8] and on risk assessment. Therefore, it is essential that adjustment factors are detailed in the screening request form [9], and each laboratory must audit them on a regular basis, making local adjustments, where appropriate [4].

#### Post-analytical recommendations

##### Risk assessment software

The software used for risk calculation must meet some minimal specifications (Table 1), in view of the variability of results across programs [10].

**Table 1:** Recommendations for the risk assessment program.

Markers should be adjusted for correction factors
Flexibility in updating local variations in distribution parameters and maternal weight, and inclusion of new markers and correction factors
Possibility of using different age curves
Possibility of adjusting for previous T21-affected pregnancy
Expression of risk at term or at the time of testing
Identification of the marker models defined for the most common aneuploidies
Easy calculation of quality indicators
Possibility of entering cfDNA and invasive test results and detailing potential outcomes (including intrauterine and perinatal fetal loss, pregnancy termination and miscarriage)
Easy data export for regional or national audits
CE marking (mandatory since 2005) in compliance with directive 98/79/CE and Royal Decree 1662/2000 regulating healthcare products for diagnosis *in vitro*

The software must allow to define different populations of pregnant women. In the unaffected population, the median of the MoM must be 1.00. Given that population standard deviation depends on the screening method employed, it is advisable that each laboratory calculates its own deviation values for each analyte. For that purpose, more than 1,000 screening results of the same laboratory must be available In the first trimester of gestation, deviations must be within the following limits: free βhCG [0.25–0.29]; PAPP-A [0.23–0.29]. If values are outside these intervals, the causes will be investigated and corrective actions will be adopted.

For women carrying trisomy-affected fetuses, the median and standard deviation will be collected from large studies, and the software program will be updated regularly.

As they are not completely independent, it is recommended that each laboratory calculates the coefficients of correlation between each pair of markers for their local population, which must be between 0.05 and 0.25 for PAPP-A with free βhCG.

It is advisable that the lower and upper truncation limits for MoM outliers be established at 0.2 and 5.0, respectively, both for free βhCG and PAPP-A. Software programs must allow to modify truncation limits, where appropriate.

##### Report of test results

Results must be reported in accordance with the needs of the local pregnant population [2], [11]. In general, it is recommended to use an online platform that allows automated data entry, which software ensures the traceability of data by the unequivocal identification of the professionals with access to the program.

Reports will include, at least, the data detailed in Table 2 and will be interpreted by the referring physician.

**Table 2:** Minimum data required in a combined screening report.

Name of the pregnant woman, date of birth, and another unequivocal identification number (medical record or social security number)
Name of the requesting physician and center
Screening test requested
Type of specimen and date of collection of the specimen
Laboratory accession code that identifies the specimen
Demographic data and information relevant to the interpretation of results (e. g. CRL, maternal age and weight)
NT measurement and units (e. g.: NT in mm)
Name and license number of the sonographer
Test results in mass units (e. g. mg/mL) and in interpretation units (e. g. MoM) adjusted for correction factors
Risk for each of the trisomies screened for

##### MoM monitoring

The laboratory can use the median values provided by the manufacturer until median values have been calculated for the local population, which requires the analysis of over 100–150 samples for each gestational week. Each laboratory must regularly audit their population medians on the basis of its level of activity. Regular audits of median MoM values will enable laboratories to verify that the deviation of ±10% from the unit is met. The identification of bias will prompt laboratories to take corrective measures and update their local MoM values.

Guidelines for biochemical processing are summarized in Table 3.

**Table 3:** Summary of recommendations for biochemical processes.

Data on gestational age, maternal weight and age, ethnicity, smoking, insulin-dependent diabetes, and ART pregnancy (with the age of the donor, where appropriate) must be reported to the laboratory specialist for a correct interpretation of biochemical test results
Blood should be collected by conventional venipuncture at the appropriate gestational age
Serum expected to be processed within 72 h of collection must be shipped and stored at 4 °C. Beyond this timeframe, the sample must be frozen at −20 °C for later processing
Freezing/thawing cycles should be avoided
The laboratory must guarantee the preanalytical and analytical conditions required for combined screening. Only analytical platforms and reagents bearing the CE marking for combined screening must be used
The total error performance must be verified by using internal and external quality control materials. Participation in the UK NEQAS cross-comparison program is recommended
It is recommended that each of the biochemical determinations required for assessing prenatal risk has been granted UNE-EN ISO 15189:2013 accreditation
The median values used for calculation of the MoM of each marker must be adjusted for the local population and revised and updated on a regular basis. On such purpose, more than 8,000 screening tests must be performed per year. Otherwise, a minimum of 2,000 screening tests is acceptable if several laboratories serving similar populations are unified when evaluating the medians of biochemical markers
Calculation software must bear the CE marking for the screening strategy performed. It is laboratory’s responsibility to be aware of and regularly check for updates to the truncation limits of biochemical markers and curves of correlation with gestational age and maternal weight
Automated entry of biochemical results minimizes transcription errors. The traceability of any modification carried out must be guaranteed by the unequivocal identification of the professionals with access to the program

### Ultrasound scanning process: quality control of ultrasonographic parameters in combined first-trimester screening

The two ultrasound parameters used in first-trimester combined screening are crown-rump length (CRL) and nuchal translucency (NT).

#### Technical and healthcare specifications

For CRL and NT to be appropriately measured, some specifications must be met in relation to the ultrasound system (mid- to high-resolution), the duration of examination (at least 25 min), the method used, and operator’s experience, who must have received specific training in first-trimester screening [12], [13].

#### Training and certification

First-trimester ultrasound should be performed by sonographers experienced and trained in the technique. In Spain, ultrasound training is included in the postgraduate training curriculum (medical residency). The Spanish Society of Gynecology and Obstetrics (SEGO) grants ultrasound specialist certification to the gynecologists who complete their residency training in accredited centers.

The SEGO Section on Ultrasonography (SESEGO) and other entities offer specific ultrasonography training courses regularly, which facilitates specialist training. The 2018 SEGO first-trimester screening guidelines recommend that a quality control of prenatal screening programs be performed on a regularly basis [14].

In Spain, quality criteria for first trimester screening ultrasound are not audited or certified by any organization. First-trimester screening quality control programs have been only implanted in a few autonomous communities.

This document contains a compilation of quality procedures described in guidelines published in USA and other European countries.

In Europe, since 1992, the *Fetal Medicine Foundation* has conducted a thorough study of first-trimester ultrasound study that has resulted in a set of technical requirements and standards. Additionally, a UK-NEQAS-certified individual certification system has been established. In USA, the American Society for Maternal-Fetal Medicine created the *Nuchal Translucency Quality Review*, a training program with similar purposes [15].

#### CRL measurement quality monitoring

##### CRL measurement standards

**Figure 1:**
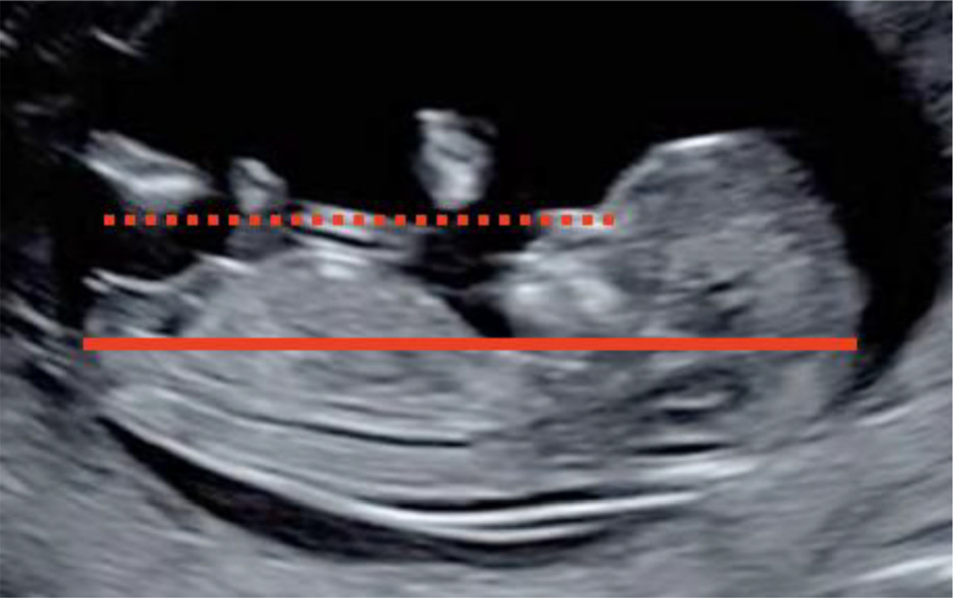
CRL measurement standards. Examination can be transvaginal or transabdominal. **CRL** between 45 and 84 mm. **Midsagittal plane:** Sagittal section of the fetus with the head in line with the body. The view must include the echogenic tip of the nose, the nasal bone if present, the diencephalon (do not include the orbit), the insertion of the umbilical cord, the bladder and the genital tubercle. The lower limbs should not be visible. **Correct visualization of the cephalic and caudal pole** with identification of the crown, the rump and the skin around them. **Neutral fetal position**, neither flexed (the pocket of amniotic fluid between the lower chin and the thorax must be equal to or greater than the width of the palate); nor extended (Fetal palate angle should be between 30° and 60° with respect to the long axis). **Orientation**: the plane of CRL should be 0–30° with respect to the horizontal so that the angle between the ultrasound beam and the CRL measurement line is 90°. To ensure that the fetal length is as close as possible to the horizontal, draw a line from the tip of the nose, which should be at the level of or above the abdominal wall with respect to the horizontal. **Magnification**: the CRL should occupy more than 60% of image space and the entire crown-rump must be seen. **Correct caliper placement**: place the calipers on the outer border of the skin on the fetal head and rump. Measure the CRL three times and report the mean of three acceptable measurements.

Ensuring strict adherence to international standards is crucial [12], [16], as shown in Figure 1.

##### CRL measurement quality control methods

While NT measurement quality control programs have been developed by health entities worldwide, the quality of CRL measurements is rarely assessed, and quality control programs are scarce.

A qualitative Image Scoring Method (ISM) similar to that of NT [17], [18], [19] has been recently proposed. However, this program is useful for training and certification, but not for large-scale auditing.

Large-scale quantitative evaluation of CRL measurement quality based on the distribution of data is less challenging, with the drawback that reference biometric data are not available for comparative analyses, as it is the case of NT. Some authors have proposed to use specific deviations of biochemical markers (PAPP-A and βhCG), which are the result of systematic CRL measurement bias [20].

##### NT measurement quality assessment methods

*NT measurement standards*: NT is the component with the highest power for aneuploidy risk assessment. This factor is strongly dependent on the operator and is subject to considerable variability that far exceeds that of biochemical markers. Therefore, adherence to a standardized NT measurement method is essential.

This is of paramount importance, as a minimal bias can negatively affect the efficacy of the screening test. As expected, inaccurate NT measurements also have a negative impact on the detection rate (DR) and the rate of false-positive results (FP). Underestimation reduces DR from 70 to 63% and FP from 2.7 to 1.2%, with overestimation having the opposite effects [21].

**Figure 2:**
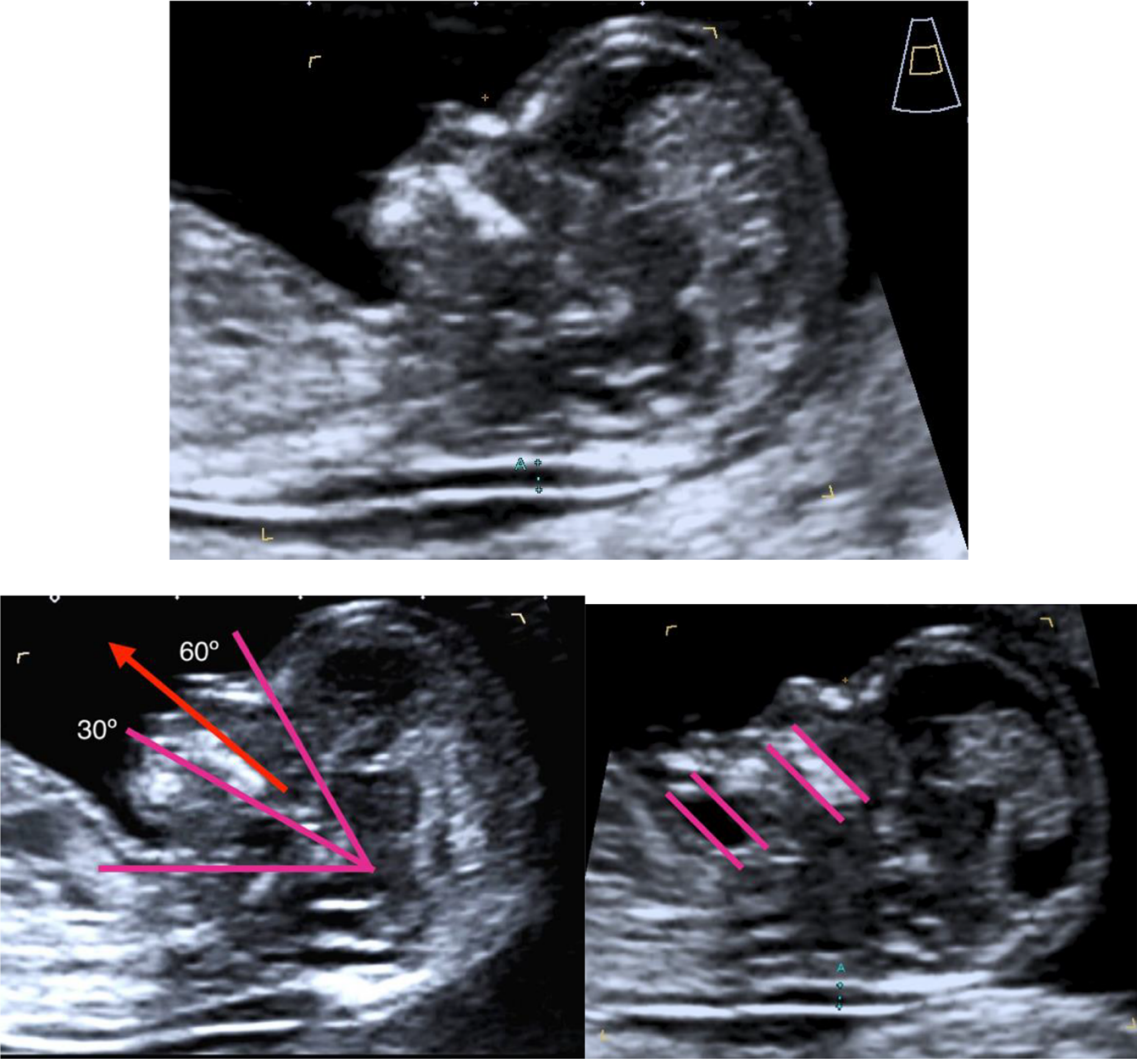
NT measurement standards. Examination can be performed via transvaginal or transabdominal route. **CRL** between 45 and 84 mm. **Magnification:** the image should only include the head and upper thorax. **Fetus in neutral position** with the head in line with the spine (hyperflexion may result in a lower NT value, whereas an extended position may increase it). Criteria for ensuring a neutral head position: 1. Fetal palate angle should be between 30° and 60° with respect to the long axis. 2. The pocket of amniotic fluid between the lower chin and the thorax must be equal to or greater than the width of the palate. **Midsagittal section** with the presence of the echogenic tip of the nose, the rectangular shape of the palate, the diencephalon, and the nuchal membrane. The alveolar bone should not be visible. Measure the NT **at the maximum** echolucent space. **Calipers on-on:** the cross of the calipers should be placed on the inner borders of the nuchal fold. **Reduce the gain** to avoid incorporating the amnion **Identification of the amniotic membrane** separated from the fetus and possible umbilical cord interposition. If the umbilical cord is around the fetal neck, use the average of the measurements of NT above and below the cord.

Figure 2 shows the optimal criteria for a correct NT measurement [12], [13], [22]

.

*NT measurement quality control methods*: Both, qualitative and quantitative methods are used to audit NT measurement quality.

In the qualitative method, a panel of reviewers uses an ISM scoring system based on a set of criteria to evaluate the quality of NT measurements [23], [24], [25]. The ISM scoring system is especially useful for initial training, or when re-training is needed after a systematic bias has been detected in an examiner. However, the application of the ISM system is time-consuming and has a high cost.

Therefore, international clinical practice guidelines recommend the use of quantitative analysis [26] or graphs, which are applicable on a large scale.

The method proposed by WIHRI (*Women & Infants Hospital of Rhode Island*) was successfully used in the multicenter FASTER study [27], [28]. This method is similar to the one used for biochemical markers and involves an analysis of statistical parameters such as the median MoM, and standard deviation from logarithmic MoM values (target: 0.08–0.13), or the percentage increase by gestational week (target: 15–35%). The median MoM is the best predictor, as it is not subject to the interference of outliers, and a MoM value ranging from 0.9 to 1.1 is considered acceptable.

A limitation of these methods is that deviations are only detected retrospectively, and feedback about sonographer’s performance is delayed, thereby hindering timely correction.

An alternative or complementary approach is the CUSUM method (*cumulative sum*) [29], [30], [31], [32]. This method is based on the assumption that a natural deviation around a target value occurs in all clinical measurements as a result of the nature of the measurement process itself, which is accepted as normal. The CUSUM method calculates the level of deviation from the expected value in each measurement process and sums it to the previous result (*S* + *t* and *S* − *t*). The highest the deviation of the mean value from the target value is, the highest the result of the cumulative sum, and the greater the deviation from the target.

The CUSUM chart is a graphical representation of the trend in the outcomes of a series of measurements over time. Sequential *S* + *t* and *S* − *t* values are represented graphically and compared against two H+ and H− thresholds (upper and lower). If the curve slopes upwards and the overestimation (or underestimation) line exceeds confidence limits, then the process is out of control. This method of ongoing evaluation has the advantage that it is a prospective method and enables early detection of bias.

##### Quality control boards

It is advisable that quality control boards (local, regional or national) are created to control the quality of first-trimester combined screening, to implement a NT measurement quality control system, and establish the most appropriate evaluation method for each setting.

A feedback mechanism should also be established for examiners to self-audit their performance. Significant deviations from the target value that persist over time should be reported to identify the cause.

### Genetic process

#### Pre-analytical recommendations

##### Information for pregnant women and professionals

Although both the Spanish Law 41/2002 [1] and Law 14/2007 of July 3rd regulating biomedical research are applicable, it is recommended that an information sheet is provided describing:

the purpose of the genetic test to which the patient is giving consent
the laboratory where the test will be performed and the destination of the biological sample after analysis
the subjects with access to test results (in case they are not anonymized)
the possibility that incidental findings may appear and patient’s option to decide whether they want to be informed or not about them
commitment to provide genetic counseling once the results are available.

Therefore, in a pre-test visit, patients must give written informed consent to undergoing the test and be informed on the limitations of the test, the future interpretation of results, and the complementary tests required.

The prescribing professional should be familiar with the test i. e. its indications, other alternative diagnostic methods, preanalytical sample collection standards, sample transport, sample refusal criteria, and know how to interpret results and the subsequent actions to be taken.

##### Pregnancy data

Apart from the basic details described in section Pregnancy details, data on the clinical indication, value of combined risk estimation, ultrasound findings, and previous history are required.

It is essential to be aware of the special circumstances in which this test is not recommendable or has a limited informative value:

the mother or the father is a carrier of a Robertsonian translocation (specify the translocation)
maternal body mass index >30 [33]
exposure to low-weight heparin [34]
ART pregnancy, which is relevant to genotyping studies [35]
vanished twin. In case of a vanished twin, a circulating cell-free DNA test (cfDNA) is not recommended
the mother is a carrier of a condition to be analyzed (included in the test) [36]
blood transfusions, organ transplant receptor, generalized infection or neoplasm in a pregnant woman, plasma therapy: All these factors may influence test results as they incorporate an indeterminate amount of plasma DNA (endogenous or exogenous).

##### Sample collection, stability, and transport conditions

Blood will be drawn by venipuncture. The collection tube and informed consent/extraction order will be identified with at least two different identifiers. It is recommended that a minimum of 6 mL of peripheral blood be collected into a vacuum tube with caution to avoid hemolysis and the sample be mixed with the anticoagulant by gently inverting the tube.

At present, there are two types of cfDNA collection tubes (although their use should be validated against the protocol and the technology available before routine use):

1. EDTA tube. This tube does not contain additive agents for the preservation of cfDNA or the prevention of cell rupture. The use of this type of tube is not recommended if the sample is not expected to be processed within 4–8 h of collection. Samples must be refrigerated (4 °C) – not frozen – for storage and processing.
2. cfDNA collection tube. This type of tube is used for preservation of cfDNA for longer periods at the temperature indicated by the manufacturer. The maximum storage time after collection must be validated analytically and indicated in the information sheet provided to the clinician [37].

Samples not meeting the quality standards established in preanalytical requirements or showing alterations (clotted, highly hemolyzed, among others) are not suitable for cfDNA testing.

##### Custody

The custody of samples should meet all general laboratory certification requirements. In view of the stability of plasma at −80 °C and of the genomic library at −20 °C, it is recommended that an aliquot of the sample be stored in these conditions for one year to meet future claims or requests for result verification.

#### Analytical recommendations

##### Methods

cfDNA testing is a recent technology for which quality standards have not yet been established. It is essential that the standard operating procedures used in the laboratory are specified, including the instruments, protocols, and associated technical staff. The cfDNA test can be used in two general contexts [38]:

coverage: whole genome at low resolution or analysis of specific regions
method of analysis: count or genotyping method.

Given the wide variety of technologies currently available, a specific methodology cannot be strictly recommended. The environmental and technological conditions for cfDNA testing must be similar to those of molecular genetic testing for prenatal diagnosis.

The algorithm for testing should have been published and preferably validated at international level, including specific data for the validated series, rate of true and false positives, sensitivity, specificity, predictive values and rate of no-call results, among other data. The algorithm must include an appropriate sample of positives and negatives for each trisomy (T21, T18 and T13) that certifies that the test has been tested in the population of interest (both external and local). It is recommended that studies in the general population have been published. When a commercial algorithm is used, it must have been granted the corresponding validation and accreditation certificates, as well as the documentation certifying that the algorithm is suitable for the methodology to be employed.

Although there is no total consensus, the threshold rate of no-call results is set at 4% of the fetal fraction (FF) in most guidelines. It is recommended that the algorithm calculates the FF as a test quality control [39] and that the FF calculation method used is reported and different from the exclusive detection of chromosome Y (e. g. genotyping or fragment calculation). The limitations of the test must also be described.

The laboratories that do not perform this test must report the laboratory where the test is performed and, where appropriate, be in possession of the corresponding documentation for control purposes.

##### Analytical quality assurance

The laboratory must have an operating procedure validation system subject to internal and external controls. At local level, both maternal plasma (with a positive or negative result for trisomy validated by an invasive method) and artificial plasma supplied by a certified manufacturer can be used. It is recommended that an annual validation protocol is implemented on a yearly basis for each of the settings to test for (at least, a test for T21, T18 and T13).

Monitoring of protocol, technical, material, and environment quality assurance methods should be performed to optimize test quality.

Entities such as GenQA (*Genomics Quality Assessment*, an UKNEQAS member) are conducting cross-comparison studies. It is an annual scheme involving two analyses performed using the technology available in each laboratory and the submission of a final report. The European study on cfDNA screening for aneuploidy is a pilot study, although in the light of the wide diffusion on European laboratories, it is expected to become a cross-comparison program throughout 2020.

#### Post-analytical recommendations

##### Interpretation of results

The test may yield the following results: HIGH RISK for one of the trisomies tested, LOW RISK for all the trisomies tested, UNINFORMATIVE or NO-CALL result.

Test results must always be interpreted by specialist staff.

A NO-CALL result may delay diagnosis as it may require the test to be repeated using the same initial plasma as the result did not pass quality controls; or require the collection of a new sample due to methodological problems (low FF, others). The recommended window for the new extraction must be indicated. In general, an UNINFORMATIVE result indicates that the test could not be appropriately performed at some stage or in some samples. The recommended subsequent action should be based on clinical evidence.

There are algorithms that combine different clinical datasets, including cfDNA, to calculate the likelihood ratio. In this case, the test can yield a numerical risk result, which must be reported along with a threshold value for high and low risk.

A note should be included to indicate that a low-risk result does not exclude the possibility of a false negative at all, and that test results should be always interpreted in relation to the results of other clinical tests. A low-risk result should always be consistent with the negative predictive values for each trisomy.

A high-risk result should always be accompanied by a positive predictive value for the corresponding trisomy. A note should also be included indicating that the test yielded a low risk for the remainder of trisomies. All high-risk results must be confirmed via an invasive technique.

If the test cannot detect complete triploidy, this needs to be indicated in the informed consent form and test results report.

##### Test results report

The test result report should include:

two sample identification numbers and date of birth
internal identification number
name of the prescribing specialist and referring center
type of sample (peripheral blood, in this case)
test and technique requested, with indication of the algorithm to be used
test results expressed as high or low risk, and actions recommended to be undertaken
FF measurement (cut-off) and, where appropriate, calculation percentage
name of the specialist(s) who issued the report
date of report
predictive values of the sample with the population of study specified.

Notification of test results must be made in a secure manner. It is recommended to use an interconnected laboratory management software, a patient portal that can be accessed by entering a username and a password or submit an anonymized and encrypted report by e-mail.

## Part II. Invasive prenatal diagnosis of genetic abnormalities

### Ultrasound-obstetric process: Invasive procedures quality control

Current strategies for prenatal screening for aneuploidy require the ultimate use of techniques that yield a definitive diagnosis and confirm high-risk results via the genetic testing of fetal material collected by an invasive technique.

This section provides a description of quality criteria for invasive testing derived from combined screening for chromosomal abnormalities.

Invasive prenatal testing must be performed by sonographers experienced and trained in the technique. There is solid evidence that unsuccessful procedures and fetal death are associated with operator’s experience [40], [41], [42] and the number of procedures performed in the center [43].

Since the emergence of prenatal cfDNA screening, the number of prenatal invasive procedures has decreased dramatically, what may entail a considerable impact on operators’ training and experience.

Sonographers must receive specific training in centers certified by healthcare institutions. To reduce the impact of the decreasing number of invasive procedures, healthcare institutions should consider changing the traditional training model based on the volume of procedures for a novel simulation-based training model, or on the centralization of invasive testing [44], [45], [46], [47]. These new training models have proven to improve skills and reduce the number of procedures required to complete training [48], [49]

A specific number of supervised procedures cannot be required for an optimization of results, and the range of the procedures required published in the literature is notably wide, between 45 and 300 amniocenteses (AC). However, skills are not expected to improve beyond 100 procedures performed independently [50], [51]. The learning curve for lowest-risk chorionic villus sampling (CVS) stabilizes from 175 procedures [52].

There is no scientific evidence supporting the establishment of a minimum number of procedures per year for an operator to maintain the acquired skills, although some institutions have arbitrarily set this number at 30 [50].

It is essential that a local, and specially an individual training and audit plan is implemented. Both, operators and patients must have easy access to these results.

Monitoring of results must be based on a set of parameters to ensure that skills and quality standards are met [50], [53]. The indicators summarized in Table 4 should be revised on a yearly basis.

**Table 4:** Record proposal for the annual evaluation of invasive procedure indicators.

Number of procedures performed
Pregnancy losses at all gestational ages
Pregnancy losses within 14 days of the procedure
Pregnancy losses within 24 weeks
Number of punctures needed
Rate of procedures that required several attempts
Rate of procedures that yielded an inadequate or insufficient sample
Rate of amniocenteses indicated after a BC due to inadequate sample
Rate of amniocenteses that yielded a hematic sample
Rate of culture failure in cytogenetic techniques after a BC or AC
Rate of complications: loss of amniotic fluid, preterm delivery, infection, bleeding, among others
Rate of anti-D prophylaxis in RhD-negative patients

Operator’s skills have been proposed to be reevaluated when the rate of fetal loss exceeds 4/100 or the rate of failure exceeds 8/100 consecutive procedures for AC or 8/100 and 5/100 respectively for CVS [50].

The indication of an invasive procedure must be considered, as a higher rate of spontaneous miscarriages unrelated to the invasive procedure is expected in the presence of certain fetal abnormalities. Regardless of the evaluation method used, the objective should be that 100% of procedures are monitored. A lower percentage is likely to result in an underestimation of the rate of pregnancy losses, as they tend to concentrate in cases lost to follow-up [54].

According to the latest systematic study published [55], the weighted risk of miscarriage after an AC is 0.91% (confidence interval of 95%, 95% CI: 0.73–1.09%). The weighted risk of AC-related fetal loss is 0.30% (95% CI: 0.11–0.49%; I2 = 70.1%).

The weighted risk of miscarriage after a CVS is 1.39% (95% CI: 0.76–2.02%). The weighted risk of losses attributable to CVS is 0.20% (95% CI: −0.13 to 0.52%; I2 = 52.7%).

### Genetic process

#### Pre-analytical considerations

##### Type of sample

All high-risk cfDNA test results should be confirmed by the analysis of free amniocytes in amniotic fluid, since this material is exclusively fetal (unlike cfDNA), which makes it possible to exclude placental confinement mosaicism. However, CVS can be an alternative in some clinical settings, as it can be performed at an earlier stage of pregnancy, despite the risk that embryonic material is analyzed (trophoblast). Thus, the type of sample will be [56]:

Amniotic fluid from week 16 (never before week 15), for all confirmation settings. A total of 5–20 mL of amniotic fluid stored in a sterilized Falcon tube (conical). Collection syringes will not be sent to the laboratory for the risk of loss of material during transport
Chorionic villi, from week 11 (never before week 10), only in cases of high risk of T21. In cases of high risk of T18 and T13, the presence of ultrasound markers suggestive of the syndrome is required because of the possibility of a placental confinement mosaicism. It is recommended that at least 2 μL of clean chorial material are collected into a sterilized Eppendorf tube containing a minimum of 1 mL of saline, phosphate-buffered saline, or a sterile culture medium to prevent tissue degradation. For CVS karyotyping, a higher volume of starting material may be required.

It is recommended that a source of maternal DNA is available (saliva, blood) to exclude, where appropriate, prenatal sample contamination with maternal material. This test is always recommended in case of CVS (at least, in female fetuses) and when hematic amniotic fluid is analyzed.

##### Stability and transport

Sample transportation using a rigid container is recommended to reduce the risk of sample tubes being crushed. If samples are shipped within 24 h of collection, they can be transported at room temperature. Otherwise, samples must be refrigerated (4–8 °C). Do not accept fetal material if it is not received within 72 h of collection for the risk of obtaining non-analyzable DNA. Do not freeze the material.

#### Genetic test indicated. Prioritization protocol

The pregnant woman must be informed on the technique that will be employed in the laboratory and provide informed consent.

##### Rapid techniques (QF-PCR/FISH)

Initially, the QF-PCR technique is recommended (fluorescence-based quantitative PCR), as it enables, where appropriate, to test for maternal contamination by a second QF-PCR on the maternal sample. It is recommended that the test covers all aneuploidies: T13, T18, T21, *X* and *Y* to confirm the risk for the three trisomies and know the fetal gender, including a maternal contamination test where appropriate. In general, a QF-PCR result should be sufficient as a diagnostic method. The recommended maximum turnaround time are 2–3 working days [57].

On suspicion of low-grade fetal mosaicism, a FISH (fluorescence in situ hybridization) analysis will be performed, as this technique can detect levels of mosaicisms below 20%. QF-PCR can detect mosaicism levels of 40% at most.

##### Genomic microarrays

It is recommended to use an array specific for prenatal diagnosis [58] with a maximum turnoaround time of 5–10 working days. A microarray result confirmatory for a trisomy is considered sufficient as a diagnostic method, regardless of the available ultrasound findings. In case that a specific trisomy is not confirmed, tests for other alterations will be performed as authorized by the informed consent, especially if the technique can detect variants of uncertain significance. Regarding mosaicisms, the rate of detection is 20–30% for comparative genomic hybridization arrays, and 10–20% for single nucleotide polymorphisms arrays.

##### G-banding

Current recommendations include a combination of QF-PCR and long-term cytogenetic culture (two weeks). In the absence of QF-PCR, long-term culture will be performed. It is recommended to carry out 2–3 separate cultures at least in two separate incubators under different conditions and culture media to prevent contamination. If an abnormal behaviour is observed within 10 natural days (7 working days), the referring specialist should be informed of a possible culture failure. If abnormality persists in the following 14 natural days (10 working days), culture failure should be reported. Ninety-five percent of karyotypes should be informed within 10 working days.

Prenatal G-banding must have a resolution of at least 400 bands for analysis. It is recommended that a minimum of 10 metaphases are revised (or more in the presence of a mosaicism-related genetic finding).

##### Prioritization protocol

First-line QF-PCR

In CVS, if the result is female and normal, a QF-PCR of maternal material will be performed. If the result is pathologic, it can be considered as a definitive diagnosis. In case a normal result not consistent with ultrasound findings is obtained, a second-line technique is recommended.

Second-line microarray/karyotype

The two techniques are equally useful in the detection of trisomies, with microarray having the advantage of a shorter turnaround time and karyotyping being able to detect rearrangements (Robertsonian translocation).

Genetic counseling, option to study parental DNA

#### Analytical quality control

The laboratory must have an operating procedure validation system, with the recommendation that both internal and external controls are performed. Entities such as GenQA provide cross-comparison studies. These studies are performed on a yearly basis for prenatal karyotype (in amniotic fluid and CVS), QF-PCR for aneuploidies and prenatal microarray.

The issuing laboratory must have proven experience in performing the election technique for prenatal use. The three techniques must yield a result in >99% of measurements.

#### Interpretation of results

The results must indicate the DETECTION or NO DETECTION, along with their clinical interpretation, given that the invasive test is diagnostic. According to best practice guidelines of genetics laboratories, the report must be provided in a specific post-test genetic counseling visit.

The implications that genetic abnormalities have for the offspring must be informed. For example, if a test is positive for T21 and T13, it is recommended that a fetal karyotype is obtained to detect the potential presence of a Robertsonian translocation and, in case of positivity, perform a parental study. Fetal karyotype analysis is not necessary if it is directly carried out in the parents to perform a heritability study.

##### Delivery of test results

The test report must include:

two sample identification numbers and date of birth
internal laboratory identification number
name of the prescribing specialist and referring center
type of sample
test and technique requested, with their limitations described
test result (including the ISCN cytogenetic formula)
interpretation of the test and recommendations on subsequent action
name of the specialist(s) who issued the report
date of report.

Notification of test results must be made in the most secure manner. It is recommended to use interconnected laboratory management software, a patient portal that can be accessed by entering a username and a password, or to submit an anonymized and encrypted report by e-mail.

#### Follow-up of results

Discordant results must be immediately reported for an audit to be performed where all supplementary documentation must be made available (whether they are false negatives or positives). For such purpose, the proposal of this consensus group is to create a national (or regional) database that includes all cases analyzed in public hospitals (an in private hospital willing to join the program) containing the following details:

test identification
record date and date of issue of the report
clinical indication, remarks
test used (indicate type: whole genome/ specific regions and counting/genotyping/other methods used)
FF measurement (YES/NO, %, genotyping method/size of fragments/other)
production model: local/outsourced
test result (low, T21, T18, T13)
laboratory-confirmed result (CVS/amniotic fluid, YES/NO)
prenatal findings (e. g. suspicion of false-negative, miscarriage, …)
date of delivery
revision of the newborn: healthy, affected
resolution and description of incidences.

## Conclusions

This consensus document aims to unify performance criteria and quality indicators (Supplementary Material, Tables 1 to 3) for the different processes of prenatal screening for aneuploidy. It is strongly recommended that a national prenatal screening strategy is established and supervised by healthcare authorities, which indicators and diagnostic procedures are regularly evaluated. Protocols should be evaluated on a regular basis to adapt to novel cost-effective technologies.

## Supplementary Material

Supplementary Material Details

Supplementary Material Details

Supplementary Material Details

## References

[1] Ley básica reguladora de la autonomía del paciente y de derechos y obligaciones en materia de información y documentación clínica.

[2] Palomaki GE, Lee JE, Canick JA, McDowell GA, Donnenfeld AE (2009). For the ACMG Laboratory Quality Assurance Committee. Technical standards and guidelines: prenatal screening for down syndrome that includes first-trimester biochemistry and/or ultrasound measurements. Genet Med.

[3] Public Health England (2107). Programme specific operating model for quality assurance of antenatal and newborn screening programmes.

[4] FMF certification of biochemical laboratories.

[5] Spencer K (2005). First trimester maternal serum screening for down’s syndrome: an evaluation of the DPC Immulite 2000 free beta-hCG and pregnancy-associated plasma protein-A assays. Ann Clin Biochem.

[6] Benn PA, Collins R (2001). Evaluation of effect of analytical imprecision in maternal serum screening for Down’s syndrome. Ann Clin Biochem.

[7] NHS fetal anomaly screening programme – screening for down’s, Edward’s and patau’s syndromes (trisomies 21,18 & 13). NHS public health functions agreement 2018–2019.

[8] Cuckle HS, Wald NJ, Thompson SG (1987). Estimating a woman’s risk of having a pregnancy associated wih Down’s syndrome using her age and serum alpha-fetoprotein level. Br J Obstet Gynecol.

[9] Chitayat D, Langlois S, Wilson RD (2011). Prenatal screening for fetal aneuploidy in singleton pregnancies. J Obstet Gynaecol Can.

[10] National NHS Down’s Syndrome Screening Programme for England. Down’s syndrome screening: risk calculation software requirements.

[11] Palomaki GE, Bradley LA, McDowell GA (2005). Down Syndrome Working Group, ACMG laboratory quality assurance Committee. Technical standards and guidelines: prenatal screening for Down syndrome. Gen Med.

[12] Salomon LJ, Alfirevic Z, Bilardo CM, Chalouhi GE, Ghi T, Kagan KO (2013). ISUOG practice guidelines: performance of first-trimester fetal ultrasound scan. Ultrasound Obstet Gynecol.

[13] (2015). GAP Exploración ecográfica del primer trimestre.

[14] (2018). GAP Cribado y Diagnóstico precoz de anomalías genéticas.

[15] Cuckle H, Platt LD, Thornburg LL, Bromley B, Fuchs K, Abuhamad A (2015). Nuchal Translucency Quality Review (NTQR) program: first one and half million results. Ultrasound Obstet Gynecol.

[16] NHSISUOG-Salomon (2013). NHS Fetal Screening Programme (NHS FASP). Recomended criteria for measurement of fetal crown rump length (CRL) as part of combined screening for Trisomy 21 within the NHS in England.

[17] Fries N, Althuser M, Fontanges M, Talmant C, Jouk PS, Tindel M (2007). Quality control of an image-scoring method for nuchal translucency ultrasonography. Am J Obstet Gynecol.

[18] Wanyonyi SZ, Napolitano R, Ohuma EO, Salomon LJ, Papageorghiou AT (2014). Image-scoring system for crown-rump length measurement. Ultrasound Obstet Gynecol.

[19] Dhombres F, Roux N, Friszer S, Bessis R, Khoshnood B, Jouannic JM (2016). Relation between the quality of the ultrasound image acquisition and the precision of the measurement of the crown-rump length in the late first trimester: what are the consequences?. Eur J Obstet Gynecol Reprod Biol.

[20] Sabria J, Guirado L, Miro I, Gomez-Roig MD, Borrell A (2017). Crown-rump length audit plots with the use of operator-specific PAPP-A and beta-hCG median MoM. Prenat Diagn.

[21] Cuckle H (2010). Monitoring quality control of nuchal translucency. Clin Lab Med.

[22] Fetal Medicine Foundation Available at.

[23] Herman A, Dreazen E, Maymon R, Tovbin Y, Bukovsky I, Weinraub Z (1999). Implementation of nuchal translucency image-scoring method during ongoing audit. Ultrasound Obstet Gynecol.

[24] Herman A, Maymon R, Dreazen E, Caspi E, Bukovsky I, Weinraub Z (1998). Nuchal translucency audit: a novel image-scoring method. Ultrasound Obstet Gynecol.

[25] Snijders RJ, Thom EA, Zachary JM, Platt LD, Greene N, Jackson LG (2002). First-trimester trisomy screening: nuchal translucency measurement training and quality assurance to correct and unify technique. Ultrasound Obstet Gynecol.

[26] Palomaki GE, Lee JE, Canick JA, McDowell GA, Donnenfeld AE, ACMG Laboratory Quality Assurance Committee (2009). Technical standards and guidelines: prenatal screening for Down syndrome that includes first-trimester biochemistry and/or ultrasound measurements. Genet Med.

[27] Malone FD, Canick JA, Ball RH, Nyberg DA, Comstock CH, Bukowski R (2005). First-trimester or second-trimester screening, or both, for Down’s syndrome. N Engl J Med.

[28] Palomaki GE, Neveux LM, Donnenfeld A, Lee JE, McDowell G, Canick JA (2008). Quality assessment of routine nuchal translucency measurements: a North American laboratory perspective. Genet Med.

[29] Biau DJ, Porcher R, Salomon LJ (2008). CUSUM: a tool for ongoing assessment of performance. Ultrasound Obstet Gynecol.

[30] Sabria J, Barcelo-Vidal C, Arigita M, Jimenez JM, Puerto B, Borrell A (2011). The CUSUM test applied in prospective nuchal translucency quality review. Ultrasound Obstet Gynecol.

[31] Hynek M, Smetanova D, Stejskal D, Zvarova J (2014). Exponentially weighted moving average chart as a suitable tool for nuchal translucency quality review. Prenat Diagn.

[32] Chang WR, McLean IP (2006). CUSUM: a tool for early feedback about performance?. BMC Med Res Methodol.

[33] Grömminger S, Erkan S, Schöck U, Stangier K, Bonnet J, Schloo R (2015). The influence of low molecular weight heparin medication on plasma DNA in pregnant women. Prenat Diagn.

[34] Ashoor G, Syngelaki A, Poon LC, Rezende JC, Nicolaides KH (2012). Fetal fraction in maternal plasma cell-free DNA at 11–13 weeks’ gestation: relation to maternal and fetal characteristics. Obstet Gynecol.

[35] Gil MM, Accurti V, Santacruz B, Plana MN, Nicolaides KH (2017). Analysis of cell-free DNA in maternal blood in screening for aneuploidies: updated meta-analysis. Ultrasound Obstet Gynecol.

[36] Grömminger S, Yagmur E, Erkan S, Nagy S, Schöck U, Bonnet J (2014). Fetal aneuploidy detection by cell-free DNA sequencing for multiple pregnancies and quality issues with vanishing twins. J Clin Med.

[37] Wong D, Moturi S, Angkachatchai V, Mueller R, DeSantis G, van den Boom D, Ehrich M (2013). Optimizing blood collection, transport and storage conditions for cell free DNA increases access to prenatal testing. Clin Biochem.

[38] Vermeesch JR, Voet T, Devriendt K (2016). Prenatal and pre-implantation genetic diagnosis. Nat Rev Genet.

[39] Gregg AR, Skotko BG, Benkendorf JL, Monaghan KG, Bajaj K, Best RG (2016). Noninvasive prenatal screening for fetal aneuploidy, 2016 update: a position statement of the American College of Medical Genetics and Genomics. Genet Med.

[40] Wijnberger LDE, van der Schouw YT, Christiaens GCML (2000). Learning in medicine: chorionic villus sampling. Prenat Diagn.

[41] Mungen E, Tutuncu L, Muhcu M, Yergok YK (2006). Pregnancy outcome following second-trimester amniocentesis: a case-control study. Am J Perinatol.

[42] Alfirevic Z (2009). Who should be allowed to perform amniocentesis and chorionic villus sampling?. Ultrasound Obstet Gynecol.

[43] Tabor A, Vestergaard CHF, Lidegaard O (2009). Fetal loss rate after chorionic villus sampling and amniocentesis: an 11-year national registry study. Ultrasound Obstet Gynecol.

[44] McWeeney DT, Schwendemann WD, Nitsche JF, Rose CH, Davies NP, Watson WJ (2012). Transabdominal and transcervical chorionic villus sampling models to teach maternal–fetal medicine fellows. Am J Perinatol.

[45] Nizard J, Duyme M, Ville Y (2002). Teaching ultrasound-guided invasive procedures in fetal medicine: learning curves with and without an electronic guidance system. Ultrasound Obstet Gynecol.

[46] Karasahin E, Alanbay I, Ercan M, Yenen MC, Dede M, Baser I (2009). Simple, cheap, practical and efficient amniocentesis training model made with materials found in every obstetrics clinic. Prenat Diagn.

[47] Wax JR, Cartin A, Pinette MG (2012). The birds and the beans: a low-fidelity simulator for chorionic villus sampling skill acquisition. J Ultrasound Med.

[48] Pittini R, Oepkes D, Macrury K, Reznick R, Beyene J, Windrim R (2002). Teaching invasive perinatal procedures: assessment of a high fidelity simulator-based curriculum. Ultrasound Obstet Gynecol.

[49] Khurshid N, Trampe B, Heiser T, Birkeland L, Duris E, Stewart K (2014). Impact of an amniocentesis simulation curriculum for training in MFM fellowship program. Am J Obstet Gynecol.

[50] Royal College of Obstetricians and Gynaecologists (2010). Amniocentesis and chorionic villus sampling. Green-top Guideline No. 8.

[51] Royal Australian and New Zealand College of Obstetricians and Gynaecologists (2020). Certification in Maternal fetal Medicine training program handbook.

[52] Saura R, Gauthier B, Taine L, Wen ZQ, Horovitz J, Roux D (1994). Operator experience and fetal loss rate in transabdominal CVS. Prenat Diagn.

[53] ISUOG Practice Guidelines (2016). Invasive procedures for prenatal diagnosis. Ultrasound Obstet Gynecol.

[54] Halliday JL, Sheffield LJ, Danks D, Lumley J (1990). Complete follow-up in assessing fetal losses after chorionic villus sampling. Lancet.

[55] Salomon LJ, Sotiriadis A, Wulff CB, Odibos A, Akolekar R (2019). Risk of miscarriage following amniocentesis or chorionic villus sampling: systematic review of literature and updated meta-analysis. Ultrasound Obstet Gynecol.

[56] Cherry AM, Akkari YM, Barr KM, Kearney HM, Rose NC, South ST (2017). Diagnostic cytogenetic testing following positive noninvasive prenatal screening results: a clinical laboratory practice resource of the American College of Medical Genetics and Genomics (ACMG). Genet Med.

[57] Association for Clinical Cytogenetics (2009). Prenatal diagnosis best practice guidelines.

[58] Suela J, López-Expósito I, Querejeta ME, Martorell R, Cuatrecasas E, Armengol L (2017). Recommendations for the use of microarrays in prenatal diagnosis. Med Clin (Barc).

