# Supplementary material for: Prenatal screening and diagnosis of genetic abnormalities: SEGO, SEQCML, AEDP consensus recommendations
Source: Adv Lab Med. 2020 Jul 27;1(3):20200043. doi: 10.1515/almed-2020-0043 (PMC10197315; doi:10.1515/almed-2020-0043)
Supplement: Supplementary file 1 — Supplementary Material Details [file j_almed-2020-0043_suppl_001.doc]

**Supplementary Table 1: Indicators for evaluation of recruitment and biochemical marker measurement in first-trimester combined screening (CS)**

| **Evaluation indicators for recruitment and biochemical testing** | | | |
| --- | --- | --- | --- |
| **Numerator** | **Denominator** | **Indicator in %** | **Standard** |
| Pregnant women recruited for combined screening (CS) | Total of pregnant women | Participation in CS | >80% |
| Screening request forms containing full demographic data | Pregnant women recruited for CS | % of missing demographic data | <5% |
| Screening requests with missing data for risk calculation | Pregnant women recruited for CS | % of screening requests with missing data for risk calculation | 0% (biochemical markers will not be validated until all necessary data are available) |
| Sample transport and storage conditions for screening | - | - | 100% of preanalytical conditions (mandatory) |
| Pregnant women undergoing first-trimester biochemical screening without NT measurement | Total pregnant women recruited for CS | % of incomplete CS | <1% without reason, such as miscarriage, legal termination of pregnancy, neural tube defects... |
| Pregnant women undergoing biochemical test by week of gestation (WG) | Total pregnant women with full CS | Recruitment by WG | Biochemical markers tested beyond the optimal WG for miscalculation of gestation (date inconsistency)  2 steps: <10%  1 step: <1% |
| Total pregnant women with full CS | Deliveries | Estimated CS coverage | >95% |
| Total analytic error in biochemically-estimated risk for the study period | - | - | <10% |
| Median of MoM for each biochemical marker for the study period | - | - | 0.9 – 1.1 |

| **Other general combined screening indicators** | | | |
| --- | --- | --- | --- |
| **Numerator** | **Denominator** | **Indicator in %** | **Standard** |
| Number of first-trimester tests that yielded a high-risk result for T21/18: specify the cut-off point | Total first-trimester tests | - | First-trimester high-risk test result |
| No. of complete screening tests by WG based on NT ultrasound | Total pregnant women with full CS | % of screening tests by WG based on NT ultrasound | - |
| Pregnancies with known outcomes | Total pregnant women recruited for CS | % pregnancies monitored until delivery | >95% |
| Pregnancies with known affected fetuses | Total pregnant women recruited for CS | % cases for evaluation | >95% |
| High-risk pregnancies with affected fetuses: True positives (TP) | Total pregnancies with affected fetuses | - | Estimated sensitivity |
| Low-risk pregnancies with unaffected fetuses: True negatives (TN) | Total pregnancies with unaffected fetuses | - | Estimated specificity |
| High-risk pregnancies with affected fetuses (TP): | Total high-risk pregnancies | - | Estimated positive predictive value |
| Diagnoses of T21,18,13 | Total pregnancies with affected fetuses | - | Estimated prevalence |
