# Supplementary material for: Prenatal screening and diagnosis of genetic abnormalities: SEGO, SEQCML, AEDP consensus recommendations
Source: Adv Lab Med. 2020 Jul 27;1(3):20200043. doi: 10.1515/almed-2020-0043 (PMC10197315; doi:10.1515/almed-2020-0043)
Supplement: Supplementary file 2 — Supplementary Material Details [file j_almed-2020-0043_suppl_002.doc]

**Supplementary Table 2. Record proposed to evaluate cfDNA test indicators (n,%)**

| - Tests performed - before first-trimester tests - in high-risk pregnancies - in pregnancies by ART (assisted reproduction) - in twin pregnancies - not indicated according current protocols (SEGO) - in women with a body mass index > 35 |
| --- |
| - No-result tests |
| - Total number and breakdown of HIGH RISK tests |
| - Rate of false positives for T21, T18 and T13. Include other (e.g. gender), where relevant |
| - Rate of true positives |
| - FALSE NEGATIVES and potential causes |
