# Supplementary material for: Prenatal screening and diagnosis of genetic abnormalities: SEGO, SEQCML, AEDP consensus recommendations
Source: Adv Lab Med. 2020 Jul 27;1(3):20200043. doi: 10.1515/almed-2020-0043 (PMC10197315; doi:10.1515/almed-2020-0043)
Supplement: Supplementary file 3 — Supplementary Material Details [file j_almed-2020-0043_suppl_003.doc]

**Supplementary Table 3. Proposal of evaluation indicators for invasive testing**

* Total pregnancy losses after chorionic villus sampling (PCVS), not only attributable to the technique.

| **Indicators applicable to invasive tests performed as a result of combined screening** | | | |
| --- | --- | --- | --- |
| **Numerator** | **Denominator** | **Indicator** | **Standard** |
| Invasive tests for high-risk pregnancy | High-risk pregnancies | % of invasive tests with indication of high risk | - |
| Pregnancy losses after chorionic villus sampling (PCVS)* | Number of CVS performed for high risk of genetic abnormality | % of PCVS pregnancy losses | <1.5% |
| Post-amniocentesis (PA) pregnancy losses** | Number of amniocenteses performed for high risk of genetic abnormality | % of PA pregnancy losses | <1% |
| Invasive tests without indication of high-risk | Low-risk pregnancies | % of invasive tests without indication of high risk | <0.5% |

** Total pregnancy losses after amniocentesis (PA), not only attributable to the technique.
